# Structural Dynamics of the Cereblon Ligand Binding Domain

**DOI:** 10.1371/journal.pone.0128342

**Published:** 2015-05-29

**Authors:** Marcus D. Hartmann, Iuliia Boichenko, Murray Coles, Andrei N. Lupas, Birte Hernandez Alvarez

**Affiliations:** Department of Protein Evolution, Max Planck Institute for Developmental Biology, Tübingen, Germany; Griffith University, AUSTRALIA

## Abstract

Cereblon, a primary target of thalidomide and its derivatives, has been characterized structurally from both bacteria and animals. Especially well studied is the thalidomide binding domain, CULT, which shows an invariable structure across different organisms and in complex with different ligands. Here, based on a series of crystal structures of a bacterial representative, we reveal the conformational flexibility and structural dynamics of this domain. In particular, we follow the unfolding of large fractions of the domain upon release of thalidomide in the crystalline state. Our results imply that a third of the domain, including the thalidomide binding pocket, only folds upon ligand binding. We further characterize the structural effect of the C-terminal truncation resulting from the mental-retardation linked R419X nonsense mutation *in vitro* and offer a mechanistic hypothesis for its irresponsiveness to thalidomide. At 1.2Å resolution, our data provide a view of thalidomide binding at atomic resolution.

## Introduction

In 1957, thalidomide was introduced as a potent sedative and anti-nausea drug, and was widely used by pregnant women to alleviate morning sickness. At the end of 1961 it was banned from the market as its catastrophic teratogenic side effects had led to horrific birth defects in more than 10.000 newborns. However, over the decades after its withdrawal, thalidomide was rediscovered as a promising anti-inflammatory, antiangiogenic and immunomodulatory agent with high potential in the treatment of a diverse spectrum of diseases including leprosy, AIDS and cancer. Especially its potential in cancer therapy sparked the search for derivatives with improved properties, which led to the establishment of a new class of immunomodulatory drugs (IMiDs). The two most relevant derivatives are lenalidomide and pomalidomide, which are both approved for the treatment of multiple myeloma.

In 2010, the protein cereblon was identified as a target of thalidomide by Ito et al. [1]. Cereblon was originally found in a genetic screen for mutations linked to mild mental retardation [2], and has been implicated in the modulation of ion channels [3], the regulation of AMP-activated protein kinase [4], and in general neural development [5]. Ito et al. showed that cereblon associates with damaged DNA binding protein 1 (DDB1) to act as a substrate receptor for the DDB1/cullin4 E3 ubiquitin ligase complex. The ubiquitin ligase activity of this complex is altered by thalidomide binding to cereblon [1], and also lenalidomide and pomalidomide were shown to bind to cereblon, competing for the same binding site [6]. In mammals, cereblon has a LON domain and a C-terminal thalidomide binding domain. However, homologs of the latter, also termed the CULT domain, are further found in animals and bacteria as single-domain proteins, that have all essential residues of the thalidomide binding site conserved [7].

**Fig 1:**
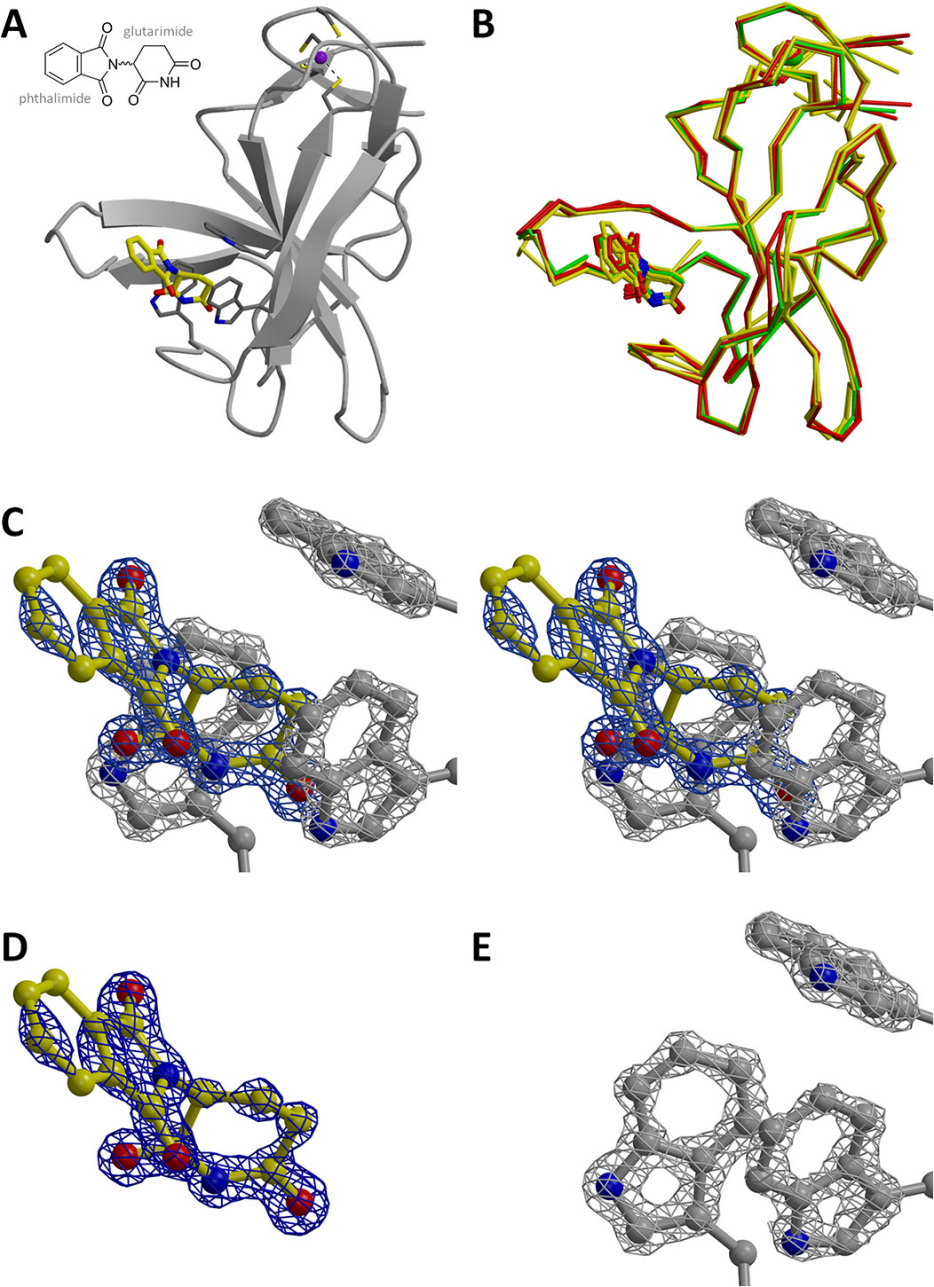
Atomic resolution structure of MsCI4•thalidomide. (A) The overall structure of MsCI4•thalidomide from the trigonal crystal form. (B) Superposition of the latter (in green) with the three MsCI4•thalidomide monomers from the orthorhombic crystal form (4V2Y, in yellow) and the two MsCI4•thalidomide monomers from the hexagonal crystal form (this work, in red). While the conformation of the protein and the glutarimide moiety is virtually identical, the orientation of the phthaloyl moieties differ by up to 13° between the structures. (C) Stereo close-up of the thalidomide molecule and the three tryptophan residues of the aromatic cage of the atomic resolution structure, together with an omit electron density map at 1.2 Å resolution. The omit map was calculated for thalidomide and the three indoles and is contoured at 6.5 sigma. Whereas individual atoms of the indoles and of the glutarimide ring are sharply localized in the density, the phthaloyl moiety is poorly resolved due to thermal disorder. (D) Only thalidomide with omit map. (E) Only the tryptophans with omit map.

The identification of cereblon as a target for thalidomide has sparked a number of structural studies. Last year, crystal structures of full-length human [8] and chicken [9] cereblon in complex with DDB1 were reported, as well as the structures of the thalidomide binding domain of mouse [8] and that of a bacterial representative, *Magnetospirillum gryphiswaldense* cereblon isoform 4 (MsCI4) [10]. These studies have shown an exceptional conservation of the domain across phyla, with a root-mean-square deviation of less than 1 Å over 100 Cα positions between the bacterial and human proteins. Architecturally, the thalidomide binding domain is a member of the β-tent fold [7], which consists of two antiparallel beta sheets that are oriented at an approximately right angle and pinned together at the top via a structural zinc ion. The thalidomide binding site is formed within the larger, C-terminal β-sheet. In most of the structures it is occupied either by thalidomide, lenalidomide or pomalidomide. These three IMiDs have a glutarimide moiety in common (see also inset in Fig 1). This moiety is bound within an aromatic cage that is mainly formed by three invariant tryptophan residues.

Now, although the thalidomide binding mode is known and invariant among animals and bacteria, evidence for natural ligands of cereblon are rather sparse. With the transcription factors Ikaros and Aiolos, the first target proteins of the cereblon/DDB1/cullin4 E3 ligase have been identified [11, 12]. The degradation of the two proteins is stimulated by thalidomide, lenalidomide or pomalidomide in myeloma cells. However, it remains unclear what exactly cereblon recognizes in absence of these agents. Given the invariant nature of the thalidomide binding site between animals and bacteria, a natural ligand universal to all domains of life seems plausible. Two kinds of such ligands have been proposed so far. One of them was strongly suggested by the nature of the binding pocket. Aromatic cages of similar architecture are typical binding sites of cationic groups like in the quaternary ammonium compounds betaine or choline [13] and are especially widespread in proteins reading posttranslationally modified lysine and arginine residues [14, 15]. However, *in vitro* binding studies of such groups to cereblon were inconclusive [8, 10]. The other potential natural ligand is uridine. Based on the binding mode of the thalidomide glutarimide ring we proposed and verified that cereblon binds uridine in the same way as thalidomide and with comparable affinity; in a co-crystal structure with MsCI4, deoxyuridine mimics the binding mode of thalidomide, and *in vivo*, in a zebrafish model system, uridine caused the same teratogenic effects as thalidomide [10]. However, although it is evidently a universal feature of cereblon, we could not consolidate a biological context for uridine recognition.

In this study, we take another step towards understanding cereblon substrate recognition by analyzing the behavior of the thalidomide binding domain in the unliganded state, based on MsCI4 as a model protein. Via X-ray crystallography, we show the domain in different conformations, and follow the unfolding of a third of the domain upon release of thalidomide in the crystalline state. Underpinned by biophysical data, our results imply that the domain as a whole only becomes structured upon ligand binding. They additionally point to the possibility that the binding site could also assume a different architecture, suitable for the binding of ligands of yet unknown nature. Further, we study the effects of premature chain termination, as caused by the mental-retardation linked R419X nonsense mutation, and find that it does not affect ligand binding. All results, mapped onto the structure of the multi-domain human cereblon, yield an intelligible picture of the structural dynamics of cereblon substrate recognition.

## Materials and Methods

### Cloning, Expression and Purification

Wild-type MsCI4 and mutant MsCI4^YW/AA^ were prepared as described [10]. The mutant MsCI4^WW/FF^ with the substitutions W36F and W59F, and mutant MsCI4^WWK/FFX^ with the additional substitution K115X were cloned with mutagenic primers using the QuickChange Site-Directed Mutagenesis Kit (Stratagene) on the basis of wild-type MsCI4 in pETHis-1a. All proteins were expressed and purified as wild-type MsCI4.

### CD spectroscopy

Circular dichroism (CD) measurements were performed using a JASCO J810 spectropolarimeter equipped with a temperature controller. Protein concentrations were 20 μM in 20 mM Tris, pH 7.6, 50 mM KF. Spectra and melting curves were recorded using a 0.1 cm path length cuvette, a bandwidth of 1 nm, and a response of 1 s. For far-UV CD spectra, a scanning speed of 100 nm/min and a data pitch of 1 nm were used. Thermal denaturation was monitored at 222 nm with a temperature ramp of 1°C per minute and a data pitch of 0.5°C. JASCO software was used for subtraction of buffer baselines, smoothing and determination of melting points.

### Fluorescence spectroscopy

Fluorescence was measured using a 2 ml cuvette in a JASCO FP-6500 spectrofluorometer at protein concentrations of 20 μM in 20 mM Tris, pH 7.5, 50 mM KF and optionally ligand concentrations of 200 μM. Samples were excited at 295 nm and emission spectra were recorded using the following settings: data pitch 0.5, excitation bandwidth 1 nm, emission bandwidth 3 nm, response 0.5 s and scan speed 200 nm/min. For data analysis, the JASCO software was used.

### Thermal shift assay (Differential scanning fluorimetry)

Ligation dependent thermal stability changes were assayed via differential scanning fluorimetry for wild-type MsCI4 and the binding deficient mutant MsCI4^YW/AA^. Samples containing 100μM protein and 400μM ligand in buffer (20 mM Tris, pH 7.5, 150 mM NaCl, 0.5 mM 2-Mercaptoethanol) supplemented with 50x SYPRO Orange dye (Sigma-Aldrich) were set up in 96-well real-time PCR plates (Thermo Scientific). The sample temperature was continuously ramped up from 25°C to 95°C at a rate of 1.25°C/min in a CFX96TM Real-Time System (Bio-Rad) monitoring the fluorescence of the dye with a temperature resolution of 0.5°C. The experiments were performed in three repeats for MsCI4 and MsCI4^YW/AA^, and five repeats for MsCI4^WWK/FFX^. The experiments for thalidomide and thymidine—including the controls without ligand—were performed in presence of 0.4% DMSO. The statistical significance of the shifts caused by the ligands was assessed with a two sample equal variance t-test.

### Crystallization, Data collection and Structure determination

Crystallization trials were performed at 294 K in 96-well sitting drop plates with 50 μl of reservoir solution and drops containing 400 nl of protein solution in addition to 400 nl of reservoir solution. The protein solution contained 3 mM thalidomide and 3% (v/v) DMSO in addition to 17 mg/ml of MsCI4 in a buffer containing 20 mM Tris, pH 7.5, 150 mM NaCl, 5 mM 2-Mercaptoethanol. In addition to the described orthorhombic crystal form [10], the screen yielded two further crystal forms, one trigonal and one hexagonal, with the crystallization conditions detailed in Table 1. Crystals of the trigonal form were loop mounted directly from the plate and flash-cooled in liquid nitrogen. For the hexagonal crystal form, crystals were transferred into a separate drop of cryo-solution prior to flash-cooling, and crystals of the orthorhombic form were washed for 40 h in a cryo-solution as indicated in Table 1. All data were collected at 100 K and a wavelength of 1 Å on a PILATUS 6M detector at beamline PXII of the Swiss Light Source (PSI, Villigen, Switzerland). Diffraction images were indexed, integrated and scaled using XDS [16]. The structures of the washed orthorhombic crystals, which contain 3 monomers of MsCI4 in the asymmetric unit (ASU), were solved on the basis of the MsCI4•thalidomide coordinates 4V2Y; the other two structures were solved by molecular replacement with MOLREP [17], locating one monomer of MsCI4 in ASU of the trigonal crystal form and 4 monomers in the ASU of the hexagonal crystal form. The structures were finalized by cyclic manual modeling with Coot [18] and refinement with REFMAC5 [19]. Data collection and refinement statistics are summarized in Table 2. All molecular depictions were prepared using MolScript [20], BobScript [21], and Raster3D [22]. The structures were deposited in the Protein Data Bank (PDB) under accession codes 5AMH (trigonal), 5AMI (orthorhombic, Wash I), 5AMJ (orthorhombic, Wash II), 5AMK (hexagonal).

**Table 1:** Crystallization conditions and cryo protection / washing procedure.

Crystal form	Reservoir solution (RS)	Cryo/washing solution
Trigonal	100 mM sodium acetate pH 4.6, 2.2 M calcium chloride	-
Orthorhombic (Wash I)	100 mM sodium acetate pH 4.6, 20%(w/v) PEG 6000	RS + 10% (v/v) PEG 300
Orthorhombic (Wash II)	100 mM tri-Sodium citrate pH 5.6, 1.0 M Ammonium phosphate	RS + 20% (v/v) PEG 300
Hexagonal	64 mM Sodium citrate pH 7.0, 100 mM HEPES pH 7.0, 10%(w/v) PEG 5000 MME	RS + 20% (v/v) PEG 300

**Table 2:** Data collection and refinement statistics.

Crystal form	Trigonal	Orthorhombic		Hexagonal
(treatment)	-	Wash I	Wash II	-
**Data collection**				
Space group	P3_2_21	P2_1_2_1_2_1_	P2_1_2_1_2_1_	P6_1_22
Cell dimensions				
*a*, *b*, *c* (Å)	51.7,51.7, 84.7	57.3, 59.6 86.7	56.7, 59.7, 88.1	137.9, 137.9, 154.4
Resolution (Å)	30.8–1.20 (1.27–1.20)*	37.3–1.75 (1.86–1.75)*	37.2–1.75 (1.86–1.75)*	39.0–2.90 (3.07–2.90)*
*R*_merge_	5.7 (74.4)	4.8 (68.2)	6.6 (47.6)	9.9 (53.2)
*I*/σ*I*	11.85 (1.78)	16.98 (1.90)	9.79 (2.16)	10.53 (2.22)
Completeness (%)	99.7 (99.3)	99.7 (98.7)	97.7 (96.5)	99.1 (98.3)
Redundancy	3.91 (3.69)	4.23 (4.23)	2.86 (2.79)	2.97 (2.95)
**Refinement**				
Resolution (Å)	30.8–1.20	37.3–1.75	37.2–1.75	39.0–2.90
No. reflections	39531	29087	28637	18776
*R*_work_ / *R*_free_	0.13 / 0.16	0.17 / 0.21	0.15 / 0.19	0.17 / 0.22
# chains / AU	1	3	3	4
**PDB code**	5AMH	5AMI	5AMJ	5AMK

*Highest resolution shell is shown in parenthesis.

### NMR Spectroscopy

NMR experiments were performed as described [10]. Significant, characteristic chemical shift changes were associated with binding of ligands employing a thalidomide-like binding mode. Typically, 1D proton spectra were acquired on 50 μM protein samples both alone and in the presence of 10–500 μM ligand. Like thalidomide, uridine induced these characteristic chemical shift changes at the lowest concentrations tested. Cytosine and thymidine did not, with concentrations of at least 500 μM. Here, this assays was repeated for the MsCI4^WWK/FFX^ mutant with thalidomide, uridine, thymidine and cytosine, yielding affinities in the same range as for wild-type MsCI4.

## Results and Discussion

### A view of thalidomide binding at atomic resolution

We previously reported the structure of MsCI4 bound to thalidomide from an orthorhombic crystal form [10]. We now obtained a new, trigonal crystal form of MsCI4•thalidomide that yielded a dataset with a resolution of 1.2 Å. The crystals contain one monomer in the asymmetric unit (ASU) in space group P3_2_21 (Fig 1A), which has the same conformation as in all other known co-crystal structures of MsCI4. This structure superimposes closely with the 3 other monomers of MsCI4•thalidomide from the orthorhombic crystal form and the 2 further new MsCI4•thalidomide monomers described below. However, between all structures, there is a significant difference in the conformation of the thalidomide molecule (Fig 1B): while the glutarimide moieties superimpose tightly, the phthaloyl moieties protruding from the binding pocket differ in their orientation by up to 13°. This angular freedom is especially apparent in the atomic resolution electron density. In an omit map calculated with missing thalidomide and tryptophan side chains in Fig 1C–1E, the individual atoms of the tryptophan side chains and of the glutarimide moiety are sharply resolved, while the density of the phthaloyl moiety shows much less detail and the most distal part shows no density at the depicted sigma level. This is a consequence of conformational disorder of the phthaloyl ring system, which is also apparent from its anisotropically refined thermal ellipsoids (not shown), which are widened in the plane of the ring system. An obvious rationale for this conformational freedom lies in the lack of specific interactions of the phthaloyl moiety with the protein. A similar degree of freedom can be derived from a superposition of the individual monomers of MsCI4 in complex with pomalidomide, lenalidomide or deoxyuridine. This underlines that there is no selective recognition for any part of the ligand apart from the glutarimide (or uracil) moiety.

### Unfolding upon ligand release in the crystalline state

**Fig 2:**
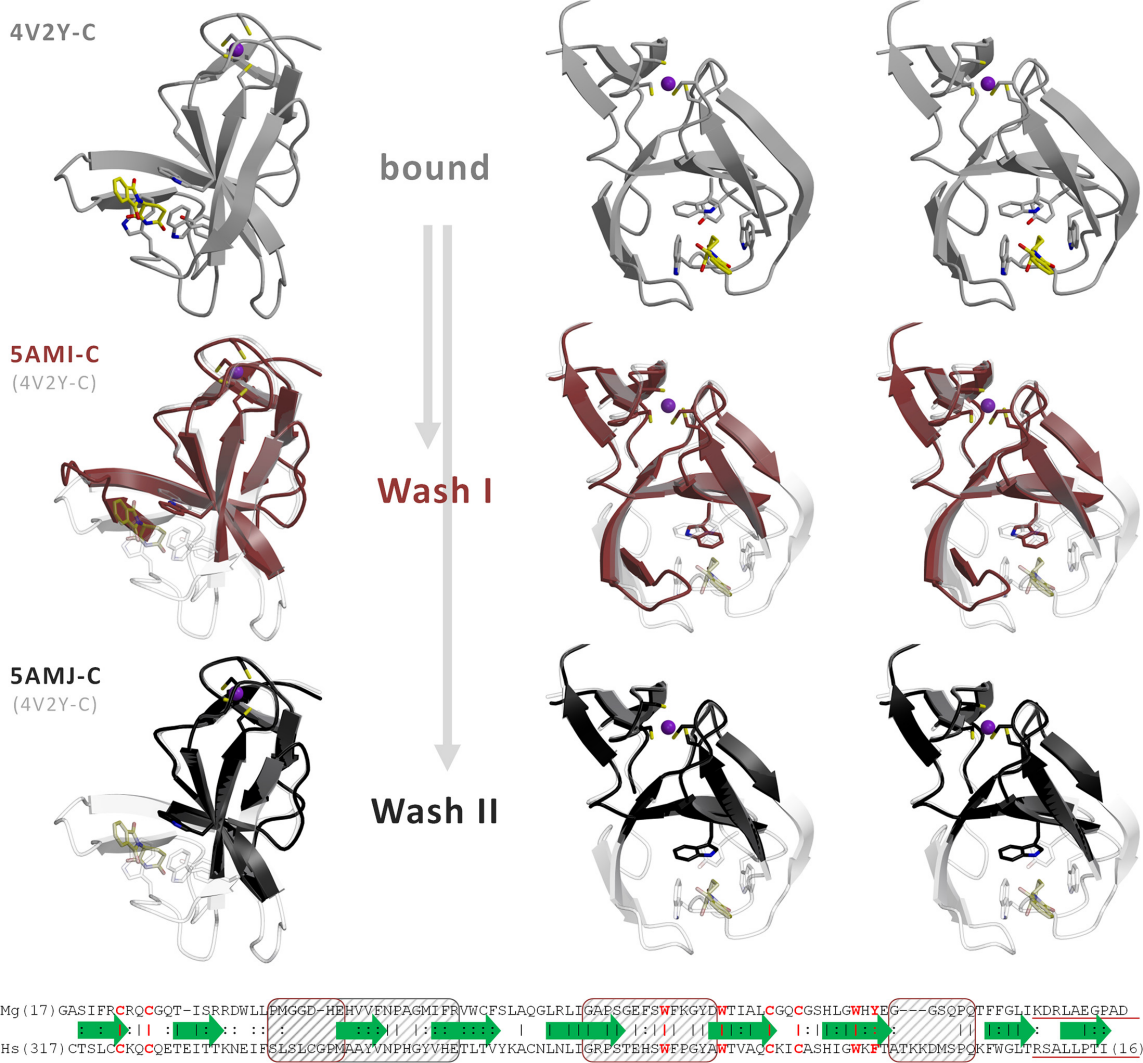
Partial unfolding of MsCI4 upon loss of thalidomide. Crystals of the orthorhombic crystal from two crystallization conditions were washed for 40 h in a solution without thalidomide. For the initial structure (grey) and both experiments, “Wash I” (brown) and “Wash II” (black), the monomer that had the ligand washed out (chain C) is depicted in two perspectives. Loss of ligand was accompanied by the unfolding of three regions. The structures after washing are overlaid with the initial structure in transparent. On the bottom, the sequence alignment of MsCI4 with human cereblon details the unfolded regions and indicates secondary structure elements. The unfolded regions are shaded. In “Wash I”, the unfolding of the first region is incomplete, so the hairpin formed by the 3^rd^ and 4^th^ β-strand is still folded, albeit shifted in position. The C-terminal segment deleted in the R419X mutant of human cereblon and the corresponding segment in MsCI4 are underlined. Key-residues are highlighted red.

To gain deeper insight into substrate binding, it was highly desirable to know the apo state of the thalidomide binding domain. As we have so far not been able to crystallize MsCI4 in the absence of ligands [10], we tried the following prolonged soaking experiment: We soaked crystals of the original orthorhombic crystal form of MsCI4•thalidomide for many hours in the cryo-protectant solution in order to “wash out” thalidomide from the binding sites. Diffraction experiments were performed with crystals from two different crystallization conditions after 40 h of washing. In the two resulting structures, thalidomide was retained in two of the three monomers in the ASU, but released in the third monomer. Strikingly, the loss of the ligand was accompanied by the unfolding of a large portion of the protein around the binding site. In one of the structures, “Wash II”, three regions totaling to 37% of the previously structured part of the main chain were no longer traceable in the electron density. The largest is the most N-terminal unfolded region, which starts between β2 and β3 and comprises the hairpin formed by β3 and β4, leaving only a C-terminal remainder of β4. The second region starts in the middle of β5, comprises the first tryptophan of the aromatic cage, and ends at the cage’s second tryptophan at the start of β6, rendering its indole side chain disordered. The third and smallest region is the loop connecting β7 and β8. In the other structure, “Wash I”, the second and third region were unfolded to the same extent, whereas the β-hairpin in the first region was still mostly structured, albeit shifted in position. The unfolding process is detailed in Fig 2.

Of the three unfolded regions, the first one does not form specific interactions with thalidomide but stabilizes the geometry of the aromatic cage and thus of the second region. In particular, it fixes the first tryptophan (W79) of the cage in its conformation by forming a hydrogen bond with the indole-side chain [10]. It further contains a highly conserved NPxG motif at the tip of the β3-β4 hairpin [7]. The second region constitutes the largest part of the thalidomide binding site, contributing two of the three tryptophans of the aromatic cage (W79 and W85) and forming hydrogen bonds to the glutarimide moiety [10]. Moreover, it contains the tyrosine residue (Y83) that was found to have an impact on cereblon function in the thalidomide-binding deficient mutant hCrbn^Y384A^ [1]. This conserved tyrosine forms hydrogen bonds with the third region and is part of a common hydrophobic core of the second and third region. It is therefore conceivable that the two regions fold and unfold in a concerted manner and that the third region, which does not directly contribute to thalidomide binding, serves to stabilize the binding site in the bound conformation. Hypothetically, an MsCI4^Y83A^ substitution corresponding to hCrbn^Y384A^ would disrupt the common hydrophobic core and therewith the mutual stabilizing interactions.

We note that in this experiment the unfolding happened within a given crystal packing, which per se restrains the conformational freedom. It would therefore be conceivable, that the extent of unfolding was limited by these restraints. However, the regions found flexible here are in perfect agreement with those identified in the following in further crystal forms of MsCI4 and mouse cereblon.

### A crystal structure with intertwined MsCI4 monomers in different conformations

**Fig 3:**
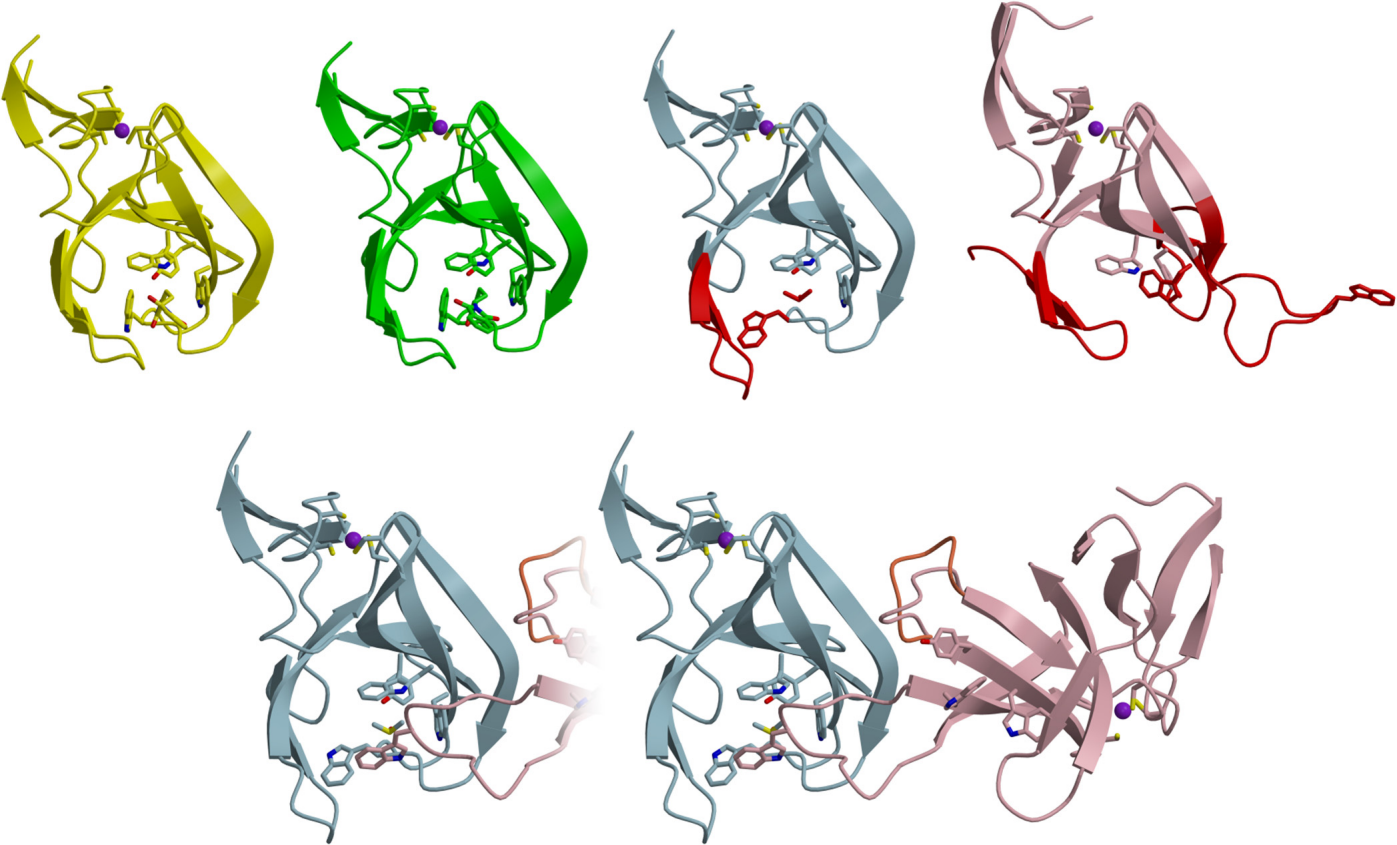
MsCI4 in different conformations in the hexagonal crystal form. Top: The 4 monomers in the ASU are found in three different conformations. The yellow and green monomer are in the known thalidomide-bound conformation. The blue monomer is in a conformation similar to the thalidomide-bound one, but its binding site is occupied by a DMSO molecule, which is accompanied with a displacement of one tryptophan of the aromatic cage (red). The pink monomer is in an overall distorted conformation and does not have the aromatic cage formed: the β3-β4 hairpin has another strand register, one of the tryptophans is flipped, and another tryptophan is displaced by 30Å (all in red). A second conformation of the 3^rd^ flexible region is displayed in another shade of red. Bottom: The blue and the pink monomers form an intertwined dimer, in which the displaced tryptophan of the pink monomer completes the aromatic cage of the blue one, trapping a DMSO molecule in a binding site with a modified architecture. Here, the second conformation of the 3^rd^ flexible region of the pink monomer is in orange.

In addition to the orthorhombic and the high-resolution trigonal crystal form, we obtained a hexagonal crystal form in a co-crystallization screen with thalidomide; we solved the structure of this third crystal form by molecular replacement, locating four monomers in the ASU. Surprisingly, only two of them are in the known thalidomide-bound conformation—the other two form an intertwined dimer and have no thalidomide bound. The two monomers of this dimer, depicted blue and pink in Fig 3, are in grossly different conformations: The blue monomer adopts an overall conformation comparable to the thalidomide-bound state but has the β3-β4 hairpin shifted in position. Therefore, the stabilizing hydrogen bond between the hairpin and the first tryptophan (W79) of the cage is broken, and the latter is found flipped out of the aromatic cage. In contrast, the conformation of the pink monomer is very different. The binding pocket is not formed and essentially all regions previously identified to be flexible are dramatically different from the thalidomide-bound conformation: In the first flexible region, the loop connecting β2 and β3 is disordered to a similar extent as in the “Wash I” structure, while the β3-β4 hairpin is shifted in position and even has another strand register. The whole second flexible region is found in an entirely different conformation—the amplitude of its largest displacement, at W79, is 30Å. Consequently, as also Y83 is not in place to anchor the second to the third region, the third flexible region is actually found in two alternative conformations, both different from the thalidomide-bound state.

Intriguingly, the W79 side chain of the pink monomer reaches over to the binding site of the blue monomer to complete the aromatic cage (Fig 3B). At the later stages of refinement, electron density of unknown origin became apparent within this cage, which was convincingly modeled as a DMSO molecule from the thalidomide stock solution. Sterically, the flipping-out of the W79 side chain from the aromatic cage of the blue monomer was a prerequisite to provide enough volume for the accommodation of DMSO. Although this particular dimeric conformation and the DMSO ligand are seemingly highly artificial, this brings to our attention that the natural ligand might be structurally quite different from thalidomide, for which the binding pocket might also adopt a conformation that is unexpectedly different from the thalidomide-bound one.

**Fig 4:**
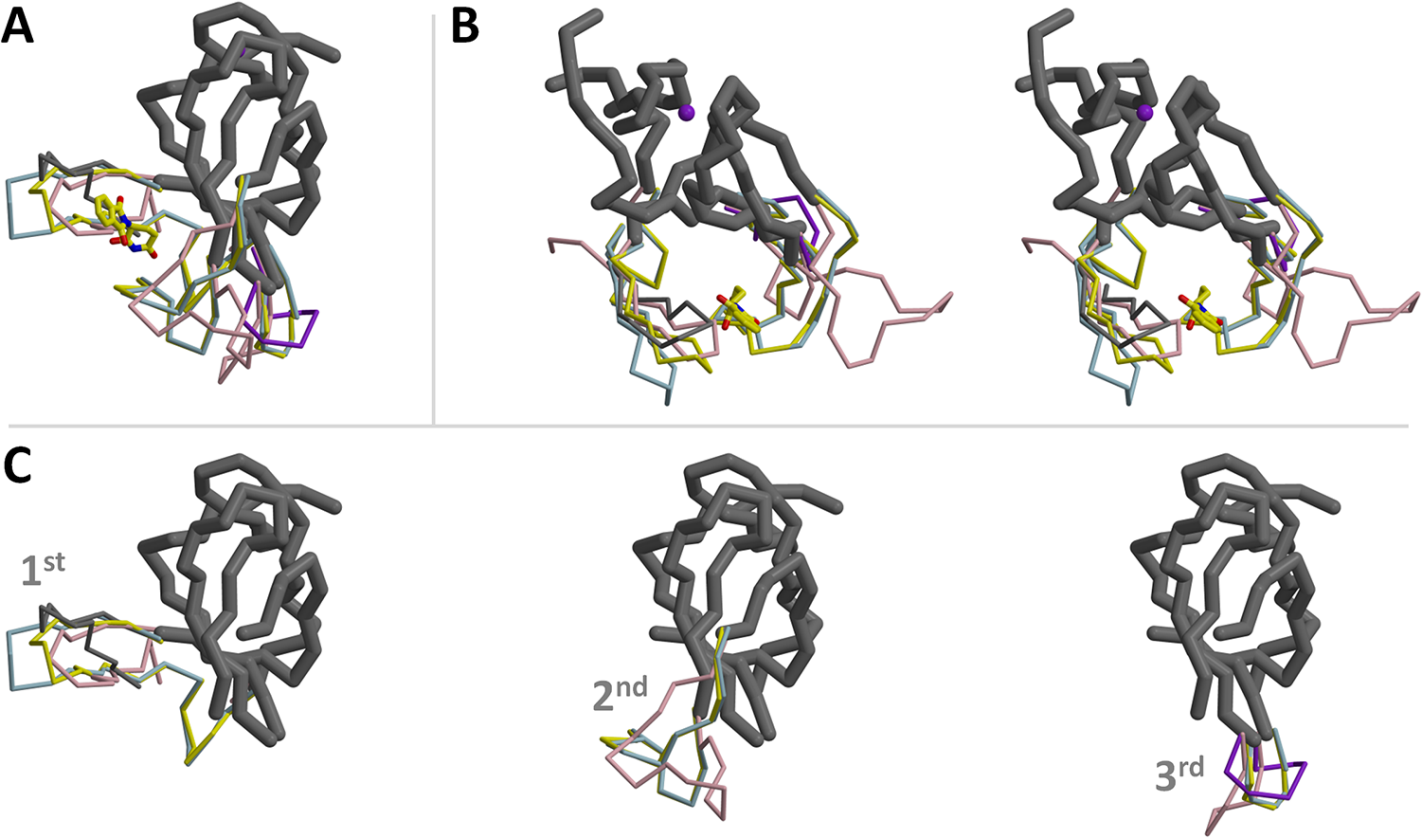
An ensemble of the available MsCI4 conformations. (A) Superposition of the thalidomide-bound conformation (yellow), the blue and pink monomers from Fig 3, the “Wash I” structure (thin dark grey), and the “Wash II” structure (thick dark grey). One alternate conformation of the pink monomer in the 3^rd^ flexible region is depicted in purple. (B) same as (A), but from the other perspective and in stereo. (C) The consensus (Wash II) structure together with the individual ensembles of the 1^st^, 2^nd^ and 3^rd^ flexible regions.

When we superimpose all available conformations of MsCI4, the thalidomide-bound one, the ones after the washing experiment, and the ones of the two monomers in the intertwined dimer, we obtain the ensemble depicted in Fig 4. Therein we find a minimal invariant consensus structure that exactly coincides with the structured part of the “Wash II” structure: While the three flexible regions derived from the washing experiment are found in a multitude of conformations, the remainder of the protein is essentially invariant in all structures.

### Folding upon ligand binding in solution

After consolidating structural evidence for immanent flexibility of the thalidomide binding domain, we set out to study the effects of ligand binding in solution. Previously, we had established an NMR-based ligand binding assay that relies on specific chemical shift changes [10]. Comparison of spectra of wild-type MsCI4 alone and in presence of thalidomide revealed significant changes for many resonances, including several prominent, upfield-shifted methyl groups. One such methyl group shifts from -0.31 to -0.89 ppm on binding of thalidomide. Chemical shift changes of this extent can be explained by close contact with aromatic moieties. However, examination of the MsCI4 binding site revealed no candidate methyl groups in contact with the aromatic rings of thalidomide itself. This indicates that ligand binding is associated considerable conformational rearrangements in residues not directly in contact with the ligand. This prompted us to further investigate the effects of ligand binding by further spectroscopic approaches.

**Fig 5:**
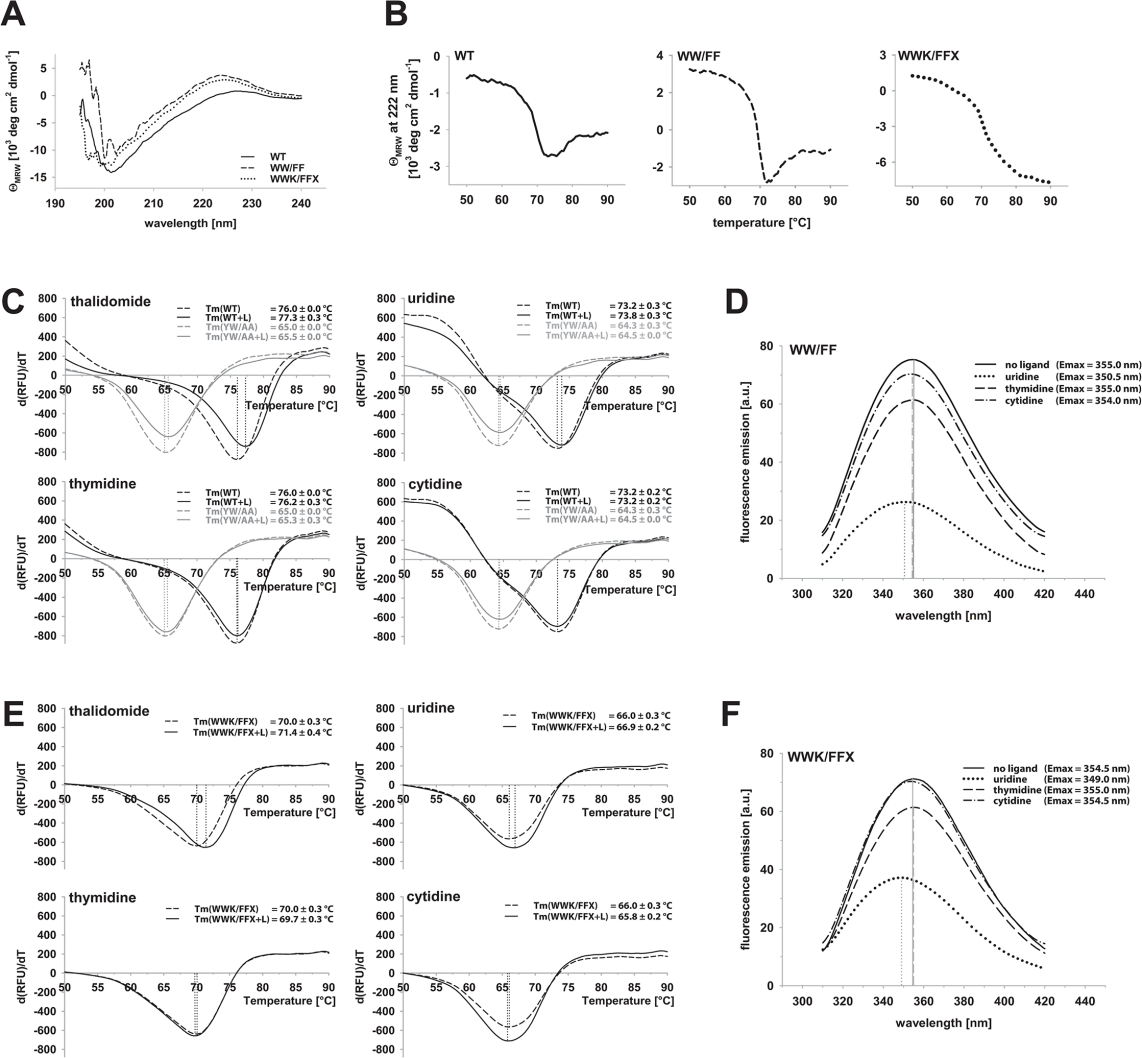
Biophysical characterization of MsCI4 and mutants. (A) Far-UV CD spectra of wild-type (WT) MsCI4 and mutants MsCI4^WW/FF^ and MsCI4^WWK/FFX^, and (B) corresponding melting curves, monitored at 222 nm. (C) Thermal stability of MsCI4 and binding deficient MsCI4^YW/AA^ in presence of thalidomide, uridine, thymidine and cytidine, as determined in the thermal shift assay. Only thalidomide and uridine lead to a shift in melting temperature with (two-tail) p-values of 1.5e-3 and 4.9e-2 as determined in a two sample equal variance t-test. (D) Tryptophan fluorescence spectra of MsCI4^WW/FF^ in presence of uridine, thymidine and cytidine. The emission maximum is shifted to shorter wavelength only in presence of the binder uridine, which can be indicative for the folding of the binding site. Note also the pronounced quenching effect indicative for binding. (E) same as (C) for the MsCI4^WWK/FFX^ mutant. As for the WT, thalidomide and uridine lead to a thermal shift, with p-values of 4.0e-4 and 1.2e-3. (F) same as (D) for the MsCI4^WWK/FFX^ mutant. The presence of uridine has the same effect as on the WT.

Firstly, we analyzed the thermal melting behavior of the protein in absence and presence of ligands. As judged by CD spectroscopy, apo MsCI4 is at least partially folded: the far-UV spectrum is indicative of secondary structure (Fig 5A), and a melting curve shows a thermal transition at about 70°C (Fig 5B). The effect of ligand binding was examined in a thermal shift assay with the known binders thalidomide and uridine, as well as the non-binders thymidine and cytidine. If the folding of the flexible regions was dependent on—and stabilized by—ligand binding, this effect should be manifest in increased thermal stability. Indeed, while thymidine and cytidine had no influence on MsCI4, thalidomide and uridine had a stabilizing effect, increasing the melting point by about 1°C. As a control, binding deficient MsCI4^YW/AA^ remained virtually unaffected by all ligands (Fig 5C).

Secondly, we monitored ligand binding via tryptophan fluorescence. For increased sensitivity we devised the mutant MsCI4^WW/FF^, in which we exchanged the tryptophan residues outside the binding site, W36 and W59, to phenylalanine. The only remaining tryptophan residues are the three of the aromatic cage, of which two became disordered in the washing experiment. An inspection of MsCI4^WW/FF^ via CD spectroscopy yielded a far UV spectrum and melting behavior comparable to the wild-type protein (Fig 5A and 5B). Finally, fluorescence emission spectra were recorded in presence of uridine, thymidine and cytidine as well as in absence of ligands. The emission maximum of apo MsCI4^WW/FF^ was found at 355 nm, which is indicative for mostly solvent exposed indole side chains, and was not shifted in presence of thymidine and cytidine. In presence of uridine, the maximum was however shifted to shorter wavelength, which can be explained by a shielding from the solvent and hence folding of the binding site (Fig 5D).

### The mental retardation-linked nonsense mutation does not affect thalidomide binding

As MsCI4 has proven a robust and reliable model system for which we have a set of useful spectroscopic techniques at hand, we employed it in a yet different scope: We reproduced the mental retardation-linked hCRBN^R419X^ nonsense mutation in MsCI4 with the corresponding K115X substitution for *in vitro* characterization. With tryptophan fluorescence studies in mind, we directly introduced the substitution in the MsCI4^WW/FF^ background, yielding the mutant MsCI4^WWK/FFX^.

The R419X mutation in hCRBN renders the protein irresponsive to thalidomide—The overall ubiquitination behavior of the E3 ligase however seems unaffected [23]. With R419 being located directly at the end of the penultimate β-strand, the premature stop effectively leads to the deletion of the last strand of the N-terminal β-sheet. It was therefore unclear whether the domain was still properly folded, which is the first question we wanted to answer with MsCI4^WWK/FFX^. After the mutant’s behavior during expression and purification was similar to the wild-type, we examined it via CD spectroscopy. The resulting far-UV spectrum was comparable to MsCI4 and MsCI4^WW/FF^ and—strikingly—the melting point was almost identical (Fig 5A and 5B). Consequently, the premature stop had seemingly no impact on the stability of the domain, so we examined its ligand binding abilities.

In analogy to MsCI4 and MsCI4^WW/FF^, we studied MsCI4^WWK/FFX^ in a thermal shift assay and via tryptophan fluorescence spectroscopy in the presence of thalidomide, uridine, thymidine and cytosine. Essentially, all results were as for full-length MsCI4: Thalidomide and uridine yielded an increase in thermal stability of about 1°C, and uridine caused a shift of the tryptophan fluorescence emission maximum to shorter wavelength, while thymidine and cytosine had no effect (Fig 5E and 5F). Therefore, ligand binding is unaffected by the premature stop, which we ultimately verified in our NMR ligand binding assay: The affinities for both thalidomide and uridine lie in the low micromolar range as determined for the wild-type protein. Consequently, the phenotype of thalidomide irresponsive hCRBN^R419X^ is apparently not due to a defect in ligand binding.

### Structural comparison to mouse cereblon

**Fig 6:**
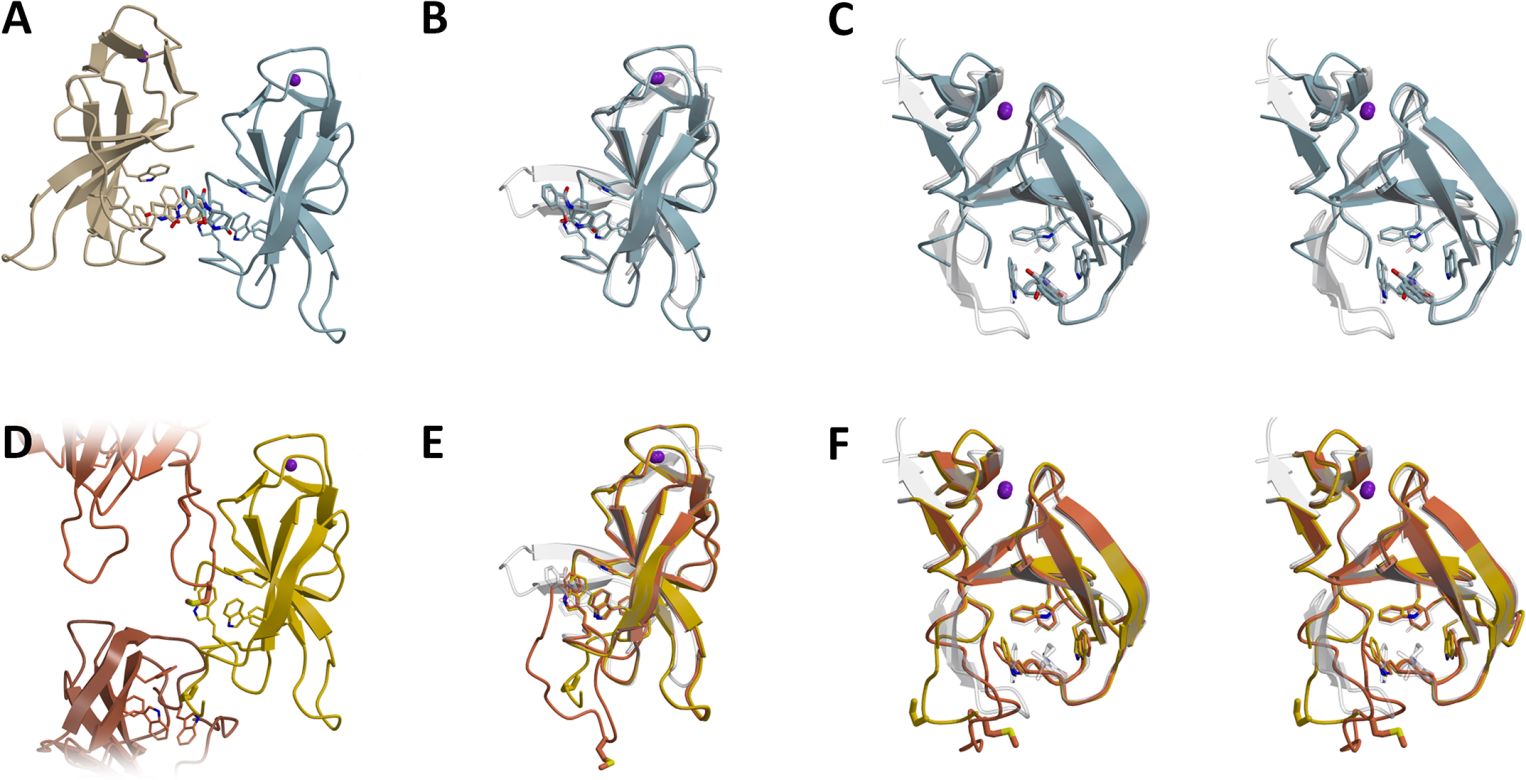
The thalidomide binding domain of mouse cereblon exhibits flexibility comparable to MsCI4. The mouse domain in complex with (blue, sand) and without thalidomide (yellow, shades of brown) is compared to MsCI4•thalidomide (transparent white). (A) Two thalidomide-bound domains from 4TZC arranged as an intertwined dimer. (B) Superposition of one monomer to MsCI4•thalidomide, illustrating the unfolded nature of the first flexible region. (C) Same as (B) but from another perspective, in stereo. (D) Apo mouse domains in 3WX2 are found in two conformations, “yellow” and “brown”, forming an endless array of interactions in the crystal lattice. (E) Superposition of both apo conformations onto MsCI4•thalidomide, showing the first flexible region in different conformations. (F) Same as (E) but from another perspective, in stereo. Note that the conformation of the tryptophans on the left is reminiscent of the flipped tryptophan in the intertwined MsCI4 dimer.

Further support for our findings on the structural dynamics is provided by the available crystal structures of the thalidomide binding domain of mouse cereblon [8]. Here, crystal structures originating from two different crystal forms show the thalidomide binding domain in several different conformations, as depicted in Fig 6.

The first crystal form contains four thalidomide-bound monomers in the ASU which form two pairs of interlaced molecules. In these pairs, the interface is formed by the thalidomide binding sites and thalidomide molecules, such that the latter form contacts with the aromatic-cages of both monomers. As a consequence, the β3-β4 hairpin cannot fold and remains mostly disordered. In this region, the otherwise identical four monomers of the ASU differ from each other: the region is disordered to different extents and the traceable parts are found in different conformations. The whole range of this flexible region matches the first one of MsCI4. Despite the missing stabilizing interaction between the β3-β4 hairpin and the first tryptophan of the cage, the binding site is in the usual thalidomide-bound conformation. This is facilitated by contacts to the thalidomide molecule of the other chain in the interlaced pairs. The neighboring thalidomide molecule forms a hydrogen bond with the tryptophan, which substitutes for the missing bond with the hairpin, and stabilizes the indole side chain in the bound conformation.

The second crystal form contains two monomers without ligands in the ASU. They adopt a conformation similar to the thalidomide-bound one. However, these two monomers also do not form the β3-β4 hairpin: the whole segment corresponding to the first flexible region is found in varying conformations. Via hydrophobic side chains from that region, the molecules stabilize the binding sites of neighboring monomers: While monomer A has the side chain of M349 stuck into the opening of the aromatic cage of monomer B, monomer B has the side chain of P348 stuck into the opening of the aromatic cage of another monomer A. In this fashion they form an endless array of intertwined molecules throughout the crystal. However, as the stabilizing hydrogen bond between the first tryptophan of the cage and the β3-β4 hairpin is lost and not substituted, the geometry of the aromatic cage is impaired. In analogy to the intertwined dimer of MsCI4 in the hexagonal crystal form, the corresponding tryptophan deviates from the thalidomide-bound conformation.

Although the conformations found in both mouse crystal forms are highly artificial, they emphasize that the flexible nature of the binding site as identified and characterized in MsCI4 is indeed not a special feature of the bacterial representative but of general significance, also for animal cereblon. As in the intertwined MsCI4 dimer, the conformation of the unliganded mouse structure is only brought about by the crystal packing.

### The thalidomide binding domain embedded in the E3 ligase complex

**Fig 7:**
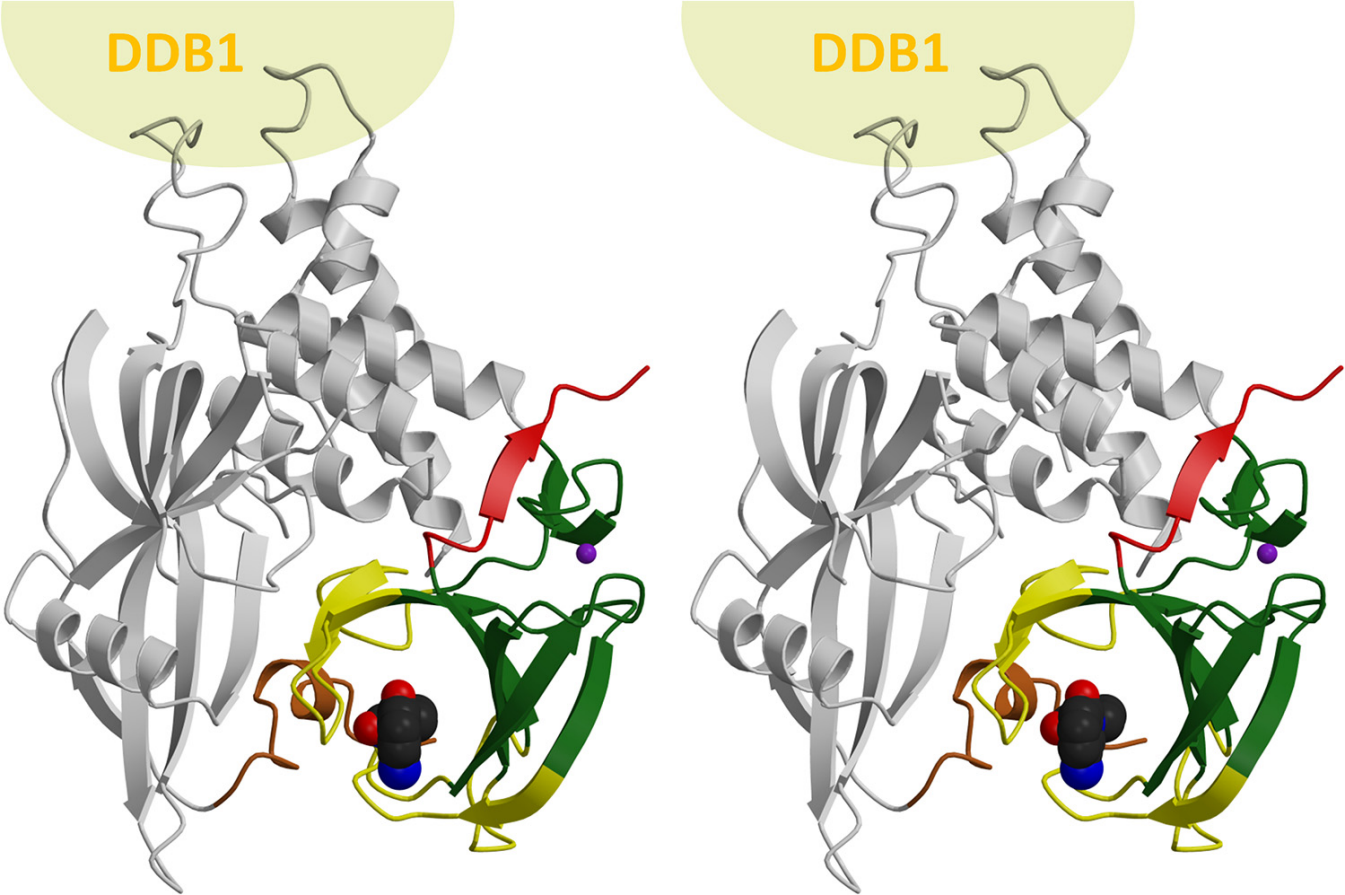
The flexible regions and mental retardation mutation in the context of human full length cereblon. At the top, interaction partner DDB1 is represented by a sphere. The thalidomide binding domain is in green (rigid core), yellow (flexible regions) and red (deleted C-terminal part in the MsCI4^WWK/FFX^ mutant). The exact boundaries of the segments are defined in Fig 2. The N-terminal extension of cereblon is in brown, the remainder of the protein grey.

To understand their functional relevance, we mapped the flexible regions onto the structure of human full-length cereblon (Fig 7). This structure [8] is bound to lenalidomide and is in a virtually identical conformation to the structures of chicken cereblon in complex with thalidomide, lenalidomide or pomalidomide [9]. Therein, the thalidomide binding domain is packed tightly to the N-terminal LON domain. A large portion of the interface between the domains is formed by the first flexible region. Further, the LON domain has a short N-terminal extension, which folds along the thalidomide binding site. As this extension, which was initially predicted to be unstructured, is exclusively in contact with the flexible regions, it seems plausible that its folding is concerted with the folding of the binding site; upon substrate binding, it would fold together with the binding site to stabilize the bound conformation, and possibly anchor the domains in their relative orientations. As determined by the PISA web server (http://www.ebi.ac.uk/pdbe/pisa/), the interface area between the domains in the fully folded, ligand-bound crystal structure amounts to 1596 Å^2^.

Reversely, in the apo state—in which the binding site would not be formed and the extension would be potentially unfolded—the interface between the domains would be significantly smaller, possibly allowing for conformational flexibility in the relative orientation of the domains: When the flexible regions are fully excluded from the calculation, the interface area between the domains is reduced by a factor of 2.6, to 609 Å^2^. A central part of this reduced interface is the region that is deleted in the retardation-linked hCRBN^R419X^ nonsense mutant (Fig 7), which mediates mostly hydrophobic contacts between the domains. When this region is omitted as well, the interface area is further reduced from 609 Å^2^ to 363 Å^2^. Hence, it is likely that the interface, and thereby the alignment of domains, is disrupted in the mutant. As we have shown that the R419X mutation does not affect ligand binding, it seems plausible that its irresponsiveness to thalidomide is due to the impaired inter-domain interface: With the thalidomide binding domain unable to dock correctly in the bound conformation, cereblon is not a functional receptor of the E3 ligase complex.

## Conclusions

This work has given insight into thalidomide binding at atomic resolution and revealed an unexpected degree of immanent structural flexibility of the thalidomide binding domain. We identify three interconnected flexible regions that fold only upon substrate binding, accounting for a third of the whole domain. A previously identified important tyrosine residue is found within the hydrophobic interface in the folded state of these regions, which explains the binding deficiency of the hCrbn^Y384A^ mutant. As deduced from the available crystal structures, the binding site can fold into a geometry deviating from the thalidomide bound one. This could imply yet further, different classes of natural ligands, possibly tertiary ammonium groups which are geometrically reminiscent of the DMSO molecule bound in one of the structures and which could be bound via cation-π interactions within the aromatic cage. Moreover, our results imply that within the E3 ligase complex, substrate binding to the thalidomide binding domain is only properly recognized when the domain is correctly docked to the remainder of the cereblon protein—which is potentially corrupted in the mental retardation linked hCrbn^R419X^ mutant. In summary, our results advance the structural basis of substrate recognition beyond a static picture and provide a mechanistic framework for the understanding of the functional role of cereblon.

## References

[1] Ito T, Ando H, Suzuki T, Ogura T, Hotta K, Imamura Y, et al Identification of a primary target of thalidomide teratogenicity. Science. 2010;327(5971):1345–50. Epub 2010/03/13. 10.1126/science.1177319 . 20223979

[2] Higgins JJ, Pucilowska J, Lombardi RQ, Rooney JP. A mutation in a novel ATP-dependent Lon protease gene in a kindred with mild mental retardation. Neurology. 2004;63(10):1927–31. Epub 2004/11/24. 15557513 10.1212/01.wnl.0000146196.01316.a2 PMC1201536

[3] Liu J, Ye J, Zou X, Xu Z, Feng Y, Zou X, et al CRL4A(CRBN) E3 ubiquitin ligase restricts BK channel activity and prevents epileptogenesis. Nature communications. 2014;5:3924 Epub 2014/05/23. 10.1038/ncomms4924 . 24845235

[4] Lee KM, Jo S, Kim H, Lee J, Park CS. Functional modulation of AMP-activated protein kinase by cereblon. Biochimica et biophysica acta. 2011;1813(3):448–55. Epub 2011/01/15. 10.1016/j.bbamcr.2011.01.005 . 21232561

[5] Chang XB, Stewart AK. What is the functional role of the thalidomide binding protein cereblon? International journal of biochemistry and molecular biology. 2011;2(3):287–94. Epub 2011/10/18. 22003441 PMC3193296

[6] Lopez-Girona A, Mendy D, Ito T, Miller K, Gandhi AK, Kang J, et al Cereblon is a direct protein target for immunomodulatory and antiproliferative activities of lenalidomide and pomalidomide. Leukemia. 2012;26(11):2326–35. 10.1038/Leu.2012.119 . 22552008 PMC3496085

[7] Lupas AN, Zhu H, Korycinski M. The thalidomide-binding domain of cereblon defines the CULT domain family and is a new member of the beta-tent fold. PLoS computational biology. 2015;11(1):e1004023 Epub 2015/01/09. 10.1371/journal.pcbi.1004023 25569776 PMC4287342

[8] Chamberlain PP, Lopez-Girona A, Miller K, Carmel G, Pagarigan B, Chie-Leon B, et al Structure of the human Cereblon-DDB1-lenalidomide complex reveals basis for responsiveness to thalidomide analogs. Nature structural & molecular biology. 2014;21(9):803–9. Epub 2014/08/12. 10.1038/nsmb.2874 . 25108355

[9] Fischer ES, Bohm K, Lydeard JR, Yang H, Stadler MB, Cavadini S, et al Structure of the DDB1-CRBN E3 ubiquitin ligase in complex with thalidomide. Nature. 2014;512(7512):49–53. Epub 2014/07/22. 10.1038/nature13527 . 25043012 PMC4423819

[10] Hartmann MD, Boichenko I, Coles M, Zanini F, Lupas AN, Hernandez Alvarez B. Thalidomide mimics uridine binding to an aromatic cage in cereblon. Journal of structural biology. 2014;188(3):225–32. Epub 2014/12/03. 10.1016/j.jsb.2014.10.010 . 25448889

[11] Gandhi AK, Kang J, Havens CG, Conklin T, Ning Y, Wu L, et al Immunomodulatory agents lenalidomide and pomalidomide co-stimulate T cells by inducing degradation of T cell repressors Ikaros and Aiolos via modulation of the E3 ubiquitin ligase complex CRL4(CRBN.). British journal of haematology. 2014;164(6):811–21. Epub 2013/12/18. 10.1111/bjh.12708 24328678 PMC4232904

[12] Kronke J, Udeshi ND, Narla A, Grauman P, Hurst SN, McConkey M, et al Lenalidomide causes selective degradation of IKZF1 and IKZF3 in multiple myeloma cells. Science. 2014;343(6168):301–5. Epub 2013/12/03. 10.1126/science.1244851 24292625 PMC4077049

[13] Dougherty DA. Cation-pi interactions in chemistry and biology: a new view of benzene, Phe, Tyr, and Trp. Science. 1996;271(5246):163–8. Epub 1996/01/12. . 8539615 10.1126/science.271.5246.163

[14] Gayatri S, Bedford MT. Readers of histone methylarginine marks. Biochimica et biophysica acta. 2014;1839(8):702–10. Epub 2014/03/04. 10.1016/j.bbagrm.2014.02.015 24583552 PMC4099268

[15] Yun MY, Wu J, Workman JL, Li B. Readers of histone modifications. Cell Res. 2011;21(4):564–78. 10.1038/Cr.2011.42 . 21423274 PMC3131977

[16] Kabsch W. Automatic Processing of Rotation Diffraction Data from Crystals of Initially Unknown Symmetry and Cell Constants. J Appl Crystallogr. 1993;26:795–800. 10.1107/S0021889893005588 .

[17] Vagin A, Teplyakov A. An approach to multi-copy search in molecular replacement. Acta Crystallogr D. 2000;56:1622–4. 10.1107/S0907444900013780 . 11092928

[18] Emsley P, Cowtan K. Coot: model-building tools for molecular graphics. Acta crystallographica Section D, Biological crystallography. 2004;60(Pt 12 Pt 1):2126–32. Epub 2004/12/02. 10.1107/S0907444904019158 . 15572765

[19] Murshudov GN, Vagin AA, Lebedev A, Wilson KS, Dodson EJ. Efficient anisotropic refinement of macromolecular structures using FFT. Acta Crystallogr D. 1999;55:247–55. 10.1107/S090744499801405x . 10089417

[20] Kraulis PJ. Molscript—a Program to Produce Both Detailed and Schematic Plots of Protein Structures. J Appl Crystallogr. 1991;24:946–50. 10.1107/S0021889891004399 .

[21] Esnouf RM. Further additions to MolScript version 1.4, including reading and contouring of electron-density maps. Acta Crystallogr D. 1999;55:938–40. 10.1107/S0907444998017363 . 10089341

[22] Merritt EA, Bacon DJ. Raster3D: photorealistic molecular graphics. Methods in enzymology. 1997;277:505–24. Epub 1997/01/01. . 18488322 10.1016/s0076-6879(97)77028-9

[23] Xu GQ, Jiang XG, Jaffrey SR. A Mental Retardation-linked Nonsense Mutation in Cereblon Is Rescued by Proteasome Inhibition. J Biol Chem. 2013;288(41):29573–85. 10.1074/jbc.M113.472092 . 23983124 PMC3795255

